# Increasing proportions of HIV-1 non-B subtypes and of NNRTI resistance between 2013 and 2016 in Germany: Results from the national molecular surveillance of new HIV-diagnoses

**DOI:** 10.1371/journal.pone.0206234

**Published:** 2018-11-08

**Authors:** Andrea Hauser, Alexandra Hofmann, Karolin Meixenberger, Britta Altmann, Kirsten Hanke, Viviane Bremer, Barbara Bartmeyer, Norbert Bannert

**Affiliations:** 1 Division of HIV and Other Retroviruses, Robert Koch Institute, Berlin, Germany; 2 Institute of Medical Virology, Charité Universitätsmedizin Berlin, Berlin, Germany; 3 Division of HIV/AIDS, STI and Blood-borne Infections, Robert Koch-Institute, Berlin, Germany; 4 Charité, Universitätsmedizin Berlin, Berlin, Germany; Institut Pasteur of Shanghai Chinese Academy of Sciences, CHINA

## Abstract

**Background:**

Molecular surveillance of newly diagnosed HIV-infections is important for tracking trends in circulating HIV-variants, including those with transmitted drug resistances (TDR) to sustain ART efficacy.

**Methods:**

Dried serum spots (DSS) are received together with the statutory notification of a new diagnosis. 'Recent infections' (<155 days) classified by a 'recent infection test algorithm' (BED-CEIA and clinical data) are genotyped in HIV-protease (PR), reverse transcriptase (RT) and integrase (INT) to determine the HIV-1 subtype, to calculate prevalence and trends of TDR, to predict baseline susceptibility and to identify potential transmission clusters for resistant variants.

**Results:**

Between January 2013 and December 2016, 1,885 recent infections were analysed regarding the PR/RT genomic region, with 43.5% of these also being subjected to the analysis of INT. The proportion of HIV-1 non-B viruses (31.3%; 591/1,885) increased from 21.6% to 36.0%, particularly the subtypes A (5.0% to 8.3%) and C (3.2% to 7.7%; all *p*_*trends*_ < 0.01). The subtype A increment is mainly due to transmissions within men who have sex with men (MSM) while subtype C transmissions are associated with heterosexuals and people who inject drugs. The prevalence of TDR was stable at 11.0% (208/1,885) over the study period. Resistances to nucleotide RT inhibitors (NRTI) and PR inhibitors (PI) were 4.5% and 3.2%, respectively, without identifiable trends. In contrast, resistances to non-NRTIs (NNRTI, 4.7%) doubled between 2014 and 2016 from 3.2% to 6.4% (p_trend_ = 0.02) mainly due to the K103N mutation (from 1.7% to 4.1%; p_trend_ = 0.03) predominantly detected in recently infected German MSM not linked to transmission clusters. Transmitted INSTI mutations were present in only one case (T66I) and resistance to dolutegravir was not identified at all. Reduced susceptibility to recommended first-line therapies was low with 1.0% for PIs, 1.3% for NRTIs and 0.7% for INSTIs, but high for the NNRTIs efavirence (4.9%) and rilpivirine (6.0%) due to the K103N mutation and the polymorphic mutation E138A. These trends in therapy-naïve individuals impact current first-line regimens and require awareness and vigilant surveillance.

## Introduction

HIV infection is still a major public health concern in EU/EEA countries with approximately 30,000 new cases reported each year [1]. Prevention by pre-exposure prophylaxis (PrEP), frequent (self-) testing of persons at risk with rapid diagnostic assays and immediate initiation of highly effective combination antiretroviral therapy (cART) following a confirmed infection are among the most promising new measures introduced in recent years to curb the number of HIV-transmissions in this region of the world [2]. However, further transmission of drug resistant viral variants that can limit the success of first-line therapy regimens remains a substantial issue [3]. Genotypic resistance testing prior to treatment initiation is therefore recommended by national and European guidelines in order to predict clinical resistance and guide the choice of individual treatments [4, 5]. The prevalence of transmitted drug resistance (TDR) is primarily linked to the cART prescription pattern in a country, the proportion of individuals treated with cART, the extend of acquired drug resistance relaying on the common therapy adherence, resistance barriers of the administered antiretroviral regimes and the frequency of virus load monitoring. As the access to cART is increasing worldwide [6], TDR requires more attention and its surveillance is gaining momentum at national and international levels [7–9].

To predict TDR in cART-naïve patients, the World Health Organization (WHO) defined relevant resistance mutations selected by protease inhibitors (PIs), nucleoside reverse transcriptase inhibitors (NRTIs) and non-NRTIs (NNRTIs). These are summarized in the WHO surveillance drug resistance mutations (WHO SDRM) list from 2009 [10]. Based on this list, the overall prevalence of TDR in Europe is reported to have been largely stable for more than a decade, affecting approximately one in ten new infections [11, 12]. This is somewhat surprising since the introduction of new drugs with higher potency and reduced tendency to induce resistance resulted in a decrease in therapy failures during this period [13]. The explanation for this apparent paradox is the high prevalence of resistance associated mutations with low impact on viral fitness that persist in transmission chains for years without selective pressure by the drug [14–16].

The surveillance of transmitted resistances of integrase strand transfer inhibtors (INSTI) is of particular interest. Raltegravir was approved in Europe in 2007, followed by elvitegravir (2012) and dolutegravir (2014). The general tolerance and the low likelihood of resistance selection due to a high genetic barrier [17] led to their widespread use and recommendation as first-line option in European guidelines [5, 18]. Since the WHO SDRM list does not include INSTI selected mutations in the last updated version of 2009, the Stanford HIV drug resistance database SDRM Worksheet for INSTI may be used instead.

In 2013 a molecular surveillance program was initiated in Germany that is based on the examination of viral sequences from recently infected among newly diagnosed HIV cases [7, 19]. The aim of the present study was to analyse the distribution of HIV-1 subtypes, the prevalence of TDR, the potential phylogenetic relationship of TDR and its impact on the baseline susceptibility in newly diagnosed patients between 2013 and 2016.

## Materials and methods

### Clinical samples

According to the “Protection Against Infection Act” (IfSG; §7) of 2001, diagnostic laboratories in Germany are obligated to report newly diagnosed HIV infections anonymously to the German public health institute (Robert Koch Institute, RKI). For surveillance programs a network of approximately eighty diagnostic laboratories (https://www.rki.de/DE/Content/InfAZ/H/HIVAIDS/Studien/InzSurv_HIV/beteiligte_Labore.html) was established that send along with the report form, residual serum from newly diagnosed HIV cases spotted onto a filter card (Whatman 903 filter paper) as dried serum spots (DSS). According to §13 of IfSG the RKI is authorized to receive blood residuals from diagnostics for surveillance purposes.

By this sampling strategy, specimens from approximately 60% of all reported newly diagnosed HIV infections are send to the RKI laboratory. Here, DSS are classified into recently acquired and long term HIV-infections according to the ECDC recommended `recent infection test algorithm´ (RITA) including results from the BED IgG Capture EIA (Sedia Biosciences Corporation, Portland, OR, USA) and clinical data from the HIV-notification database (CDC classification, CD4 cell count and viral load) [19]. Only recent infections are subsequently processed for HIV-genotyping. Sequence data are finally merged with the provided anonymous socio-demographic data from the HIV-notification form (gender; transmission routes: men who have sex with men (MSM), persons with heterosexual contact (HET), people who inject drugs (PWID); regions of patient origin) for analysis. The data protection officer of the Robert Koch Institute and the Federal Commissioner for Data Protection and Freedom of Information approved the study protocol (III-401/008#0016).

### HIV-1 subtyping, drug resistance interpretation and phylogenetic analysis

HIV-1 genotypes from the protease (PR) and reverse transcriptase (RT) genomic region (2013–2016) and the integrase (INT) genomic region (2014–2016) were generated according to the previously published protocols [20, 21]. Since 2015, Sanger sequencing has been substituted by next generation sequencing (NGS) using the Illumina MiSeq platform as described previously [22]. After extensive evaluations, a 20% threshold for defining ambiguities was applied to the NGS generated sequences to maintain consistency (S1 Text).The HIV-1 subtype was assigned by applying the REGA HIV Subtyping Tool (3.0) [23] and COMET HIV-1 (1.0) [24] to the *pol*-sequence. In cases where a subtype or circulating recombinant form (CRF) could not be assigned, a maximum-likelihood tree with bootstrap (IQ-TREE 1.5.5) was calculated using the HIV-1 subtype reference panel from the Los Alamos HIV sequence data base. Only subtype classifications based on bootstrap values of >70% in the tree topology were taken into account, otherwise they were classified as unique recombinant form (URF).

The prevalence of TDR was calculated from the number of persons infected with viral variants carrying at least one mutation included in the WHO SDRM list [10]. Transmitted INSTI mutations were defined as the detection of mutations listed in the Stanford HIV drug resistance database SDRM Worksheet for INSTI (https://hivdb.stanford.edu/pages/SDRM.worksheet.INI.html; updated in June 2016). Phenotypic resistance was predicted using the Stanford HIV Drug Resistance Database 8.4 algorithm (Stanford HIVbd) [25]. Three levels of resistance were scored: (i) S = susceptible (including susceptible and potential resistant levels) (ii) I = intermediate (including low and intermediate levels) and (iii) R = resistant (high level resistance). Predictions of primary resistance to recommended and alternative first-line therapy options are based on the EACS treatment guidelines 9.0 [5] which include the NNRTIs efavirenz and rilpivirine, the NRTIs abacavir, lamivudine, tenofovir and emtricitabine, the PIs atazanavir, darunavir and lopinavir and the INSTIs raltegravir, elvitegravir and dolutegravir.

Drug resistance mutations present in a proportion of ≥ 0.5% in the dataset were defined to be `frequent mutations´ and were used for trend analysis. In addition, sequences carrying one of the `frequent mutations´ were applied to phylogenetic analysis to allow the spread of resistance mutations within transmission networks to be mapped. For this purpose sequences were aligned with 33 reference sequences from the Los Alamos database and trimmed to 1026 base pairs. To select the optimal tree model, Maximum Likelihood (ML) phylogenies were reconstructed using the Ultrafast Bootstrap approximation in IQ-TREE with 10,000 replicates with the integrated model selection algorithm [26, 27]. A group O sequence was used as outgroup for the ML tree reconstruction. The tool `Transmic´ (https://github.com/kavehyousef/code) [28] was used to identify clusters of closely related sequences, possibly linked by direct transmission or very short transmission chains (putative transmission clusters). Therefore, a 99% bootstrap cut-off and a 4.5% mean pairwise patristic distance were used as a cluster threshold as reported by other groups [29, 30]. The tree was visualized in Figtree (version 1.4.0).

### Statistics

Statistical analyses were performed using STATA (version 14.2). Continuous variables were analyzed using median and interquartile range (IQR). The chi^2^ test was used for bivariate comparison and logistic regression to assess the odds ratios (OR) and 95% confidence intervals (CI). Changes in the prevalence over time were analyzed using the chi^2^ test for trend of odds.

## Results

### Characterisation of the study population

Between 2013 and 2016, a total of 10,643 DSS of newly diagnosed HIV-cases were submitted to the RKI along with the anonymous report and 3,380 (31.8%) were classified as recent infections. From these we were able to obtain 1,885 (55.8%) HIV-1 genotypes of the PR and RT genomic regions. The median plasma viral load was 140,500 copies/ml (IQR 32,269–991,106), the median CD4 cell count was 454 cells/μl (IQR 304–612) and the median age of the newly diagnosed individuals was 36.3 years. Baseline patient characteristics are shown in Table 1. During the study period the most remarkable change was an increase in the proportion of HET (7.6% to 13.1%) and persons with African origin (3.2% to 7.5%) between 2013 and 2015 followed by a slight decline in 2016 (to 11.4% and 6.0%, respectively).

**Table 1:** Baseline characteristics of the study population.

		Protease/Reverse Transcriptase					Integrase
	n	2013–2016 (n = 1,885) %	2013 (n = 278) %	2014 (n = 466) %	2015 (n = 624)%	2016 (n = 517) %	2014–2016 (n = 820) %
**Gender**							
Male	1,638	86.9	87.8	88.6	87.0	84.7	87.2
Female	228	12.1	11.5	10.7	12.3	13.4	11.8
Not reported	19	1.0	0.7	0.6	0.6	1.9	1.0
**Mode of transmission**							
MSM	1,091	57.9	60.8	62.5	55.6	54.9	55.5
HET	207	11.0	7.6	9.7	13.1	11.4	10.7
PWID	71	3.8	4.0	2.2	4.3	4.5	5.2
Other	7	0.4	0.7	0.4	0.3	0.2	0.4
Not reported	509	27.0	27.0	25.3	26.6	29.0	28.2
**Region of origin**							
Germany	1,147	60.9	59.4	60.7	56.6	66.9	61.3
Eastern Europe	59	3.1	2.5	4.1	3.2	2.5	2.9
Western Europe	60	3.2	4.7	2.6	4.2	1.7	3.3
Central Europe	77	4.1	3.2	4.7	3.7	4.5	4.8
Asia/Oceania	36	1.9	1.4	2.4	2.9	0.6	1.7
America/Caribbean	46	2.4	2.5	2.2	3.2	1.7	2.4
Africa	111	5.9	3.2	5.2	7.5	6.0	6.1
Other	8	0.4	0.4	0.2	0.5	0.6	0.5
Not reported	341	18.1	22.7	18.0	18.3	15.5*	17.0

MSM: men who have sex with men; HET: persons with heterosexual mode of transmission; PWID: people who inject drugs

* *p* <0.05.

HIV-1 genotypes of the INT genomic region were available for 820 of the 1,885 DSS (43.5%) from newly diagnosed cases in 2014 (70/466, 16%), 2015 (348/624, 57%) and 2016 (402/517, 79%). The proportional distribution in subgroups was congruent between the total study population (n = 1,885) and the subset (n = 820) (Table 1).

### Prevalence and trends of HIV-1 subtypes

**Fig 1:**
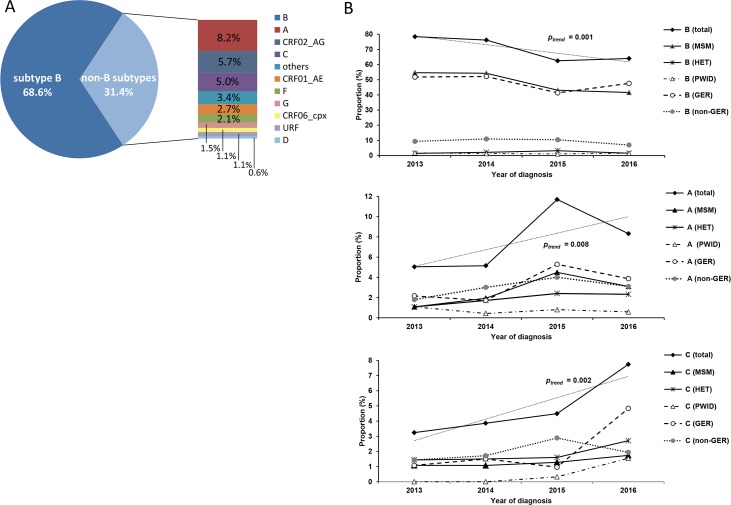
Analysis of HIV-1 subtypes. (A) Proportion of HIV-1 subtypes in the study population. (B) Trend analysis for major subtypes that show a significant increase or decrease (p < 0.05) between 2013 and 2016. Trend lines and *p-*values are indicated for the total proportions of subtypes. MSM: men who have sex with men; HET: persons with heterosexual mode of transmission, PWID: people who inject drugs, GER: German.

Subtype B infections were predominant in the total study population with 68.6% (CI95% 66.5–70.7; 1,294/1,885) and primarily affected MSM and persons of German origin (Fig 1, Table 2). The proportion of subtype B infections was significantly decreasing from 78.4% in 2013 to 64.0% in 2016 (*p*_*trend*_ = 0.001). This trend is primarily based on the two above mentioned subgroups and can also be observed in non-Germans (all *p*_*trend*_ > 0.05) (Fig 1B).

**Table 2:** Analysis of infections with major HIV-1 subtypes regarding transmission groups and regional origin.

	Subtype B (n = 1,294) %	Subtype A(n = 154) %	CRF02_AG(n = 108) %	Subtype C (n = 95) %
MSM	68.7	36.4	22.2	26.3
PWID	2.0	8.4	7.4	10.5
HET	3.3	24.7	32.4	36.9
Not reported/ other	26.0	30.5	38.0	26.3
				
Germany	68.9	43.5	32.4	43.2
Not Germany	13.7	39.0	44.4	42.1
Not reported	17.4	17.5	23.2	14.7

MSM: men who have sex with men; HET: persons with heterosexual mode of transmission, PWID: people who inject drugs

Among the non-B infections (31.4%, CI95% 29.3–33.5, 591/1,885) the most prevalent were subtype A (8.2%), CRF02_AG (5.7%) and subtype C (5.0%) (Fig 1A). Almost half of the infected individuals were of German origin, while the other half was of foreign origin (Table 2). Subtype A infected persons of foreign origin (n = 60) mostly originated from Central/East Europe (n = 31) or Sub-Saharan Africa (n = 13) and subtype C or CRF02_AG infected persons of foreign origin (n_C_ = 40 and n_AG_ = 48) largely originated from Sub-Saharan Africa (n_C_ = 26 and n_AG_ = 21) (Table 2). The increment in the proportion of non-B infections is mostly due to a significant increase of subtype A (*p*_*trend*_ = 0.008) and C (*p*_*trend*_ = 0.002) (Fig 1B). While subtype A peaked in 2015 and declined somewhat in the following year, subtype C proportions increased steadily and reached a maximum in 2016. These developments are also evident in most of the analysed subgroups except for subtype C in non-Germans, which peaked in 2015 (Fig 1B).

### Prevalence and trends of TDR

**Fig 2:**
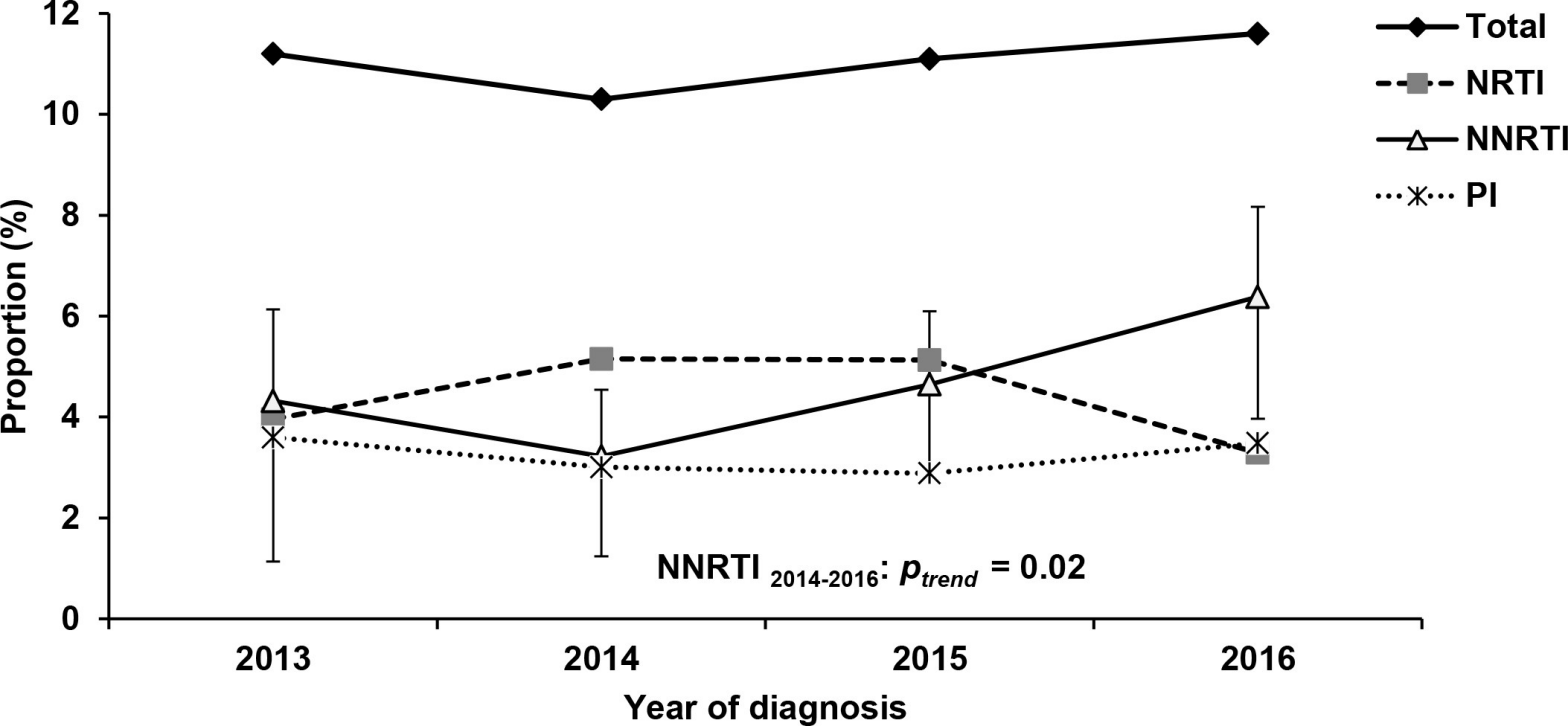
Trends of transmitted drug resistance among newly diagnosed HIV cases with recent HIV infections in total and according to drug classes. The confidence interval for NNRTI resistance is indicated.

The overall prevalence of TDR according to the WHO SDRM list was 11.0% (CI95% 9.7–12.5) (Table 3). There was little variation in the four year period between 2013 and 2016 (*p*_*trend*_ = 0.68) (Fig 2). Mono resistance was present in 9.9% of the patients (NNRTI: 3.6%, NRTI: 3.4%, PI: 2.8%), dual and triple class resistance in 1.0% and 0.2%, respectively. Taking mono and multiclass TDR together (cumulative counts), NNRTI resistance was most frequently found with 4.7% (CI95% 3.9–5.8), followed by NRTI resistance (4.5%, CI95% 3.6–5.5), and PI resistance (3.2%, CI95% 2.5–4.1) (Table 3). A trend analysis revealed that NRTI resistance decreased to its lowest levels in 2016 (*p*_*trend*_ = 0.47), while NNRTI resistance increased within the study period (*p*_*trend*_ = 0.06). This increase was significant between 2014 and 2016 (*p*_*trend*_ = 0.02). PI resistance did not show any tendency to increase or decrease between 2013 and 2016 (*p*_*trend*_ = 0.99) (Fig 2).

**Table 3:** Prevalence of TDR within subgroups between 2013 and 2016.

	Over all % (n)	Mode of transmission				Region of Origin						Subtypes			
		MSM % (n)	HET % (n)	PWID % (n)	Not rep. /other % (n)	GER % (n)	EUR % (n)	AM % (n)	AFR % (n)	AS % (n)	Not rep. /other % (n)	A % (n)	B % (n)	C % (n)	CRF 02_AG % (n)
**TDR**	11.0 (208)	11.4 (124)	6.8 (14)	5.6 (4)	12.8 (66)	11.2 (128)	5.6 (11)	13.0 (6)	12.6 (14)	5.6 (2)	13.5 (47)	14.9 (23)	12.4 (160)	8.4 (8)	6.5 (7)
**NRTI**	4.5 (84)	4.4 (48)	3.4 (7)	2.8 (2)	5.2 (27)	4.4 (51)	2.6 (5)	4.3 (2)	6.3 (7)	2.8 (1)	5.2 (18)	1.9 (3)	5.7 (74)	3.2 (3)	1.9 (2)
**NNRTI**	4.7 (89)	4.9 (53)	4.3 (9)	1.4 (1)	5.0 (26)	4.4 (51)	3.1 (6)	13.0 (6)	5.4 (6)	5.6 (2)	5.2 (18)	5.2 (8)	4.9 (63)	5.3 (5)	5.6 (6)
**PI**	3.2 (60)	3.7 (40)	0.5 (1)	1.4 (1)	3.5 (18)	3.5 (40)	1.0 (2)	0.0 (0)	2.7 (3)	2.8 (1)	4.0 (14)	9.1 (14)	3.2 (41)	2.1 (2)	0.9 (1)

MSM: men who have sex with men; HET: persons with heterosexual mode of transmission; TM: mode of transmission; rep.: reported; GER: Germany; EUR: Europe; AM: America; AFR: Africa; AS: Asia

Significantly higher proportions of TDR were found in MSM compared to HET (*p* = 0.03) and in Germans compared to non-Germans (33/397, 8.3%; *p* < 0.13). However, the prevalence was also high in persons with American and African origin, but the total number of cases with this origin was low (n = 6 and 14, respectively) (Table 3). Moreover, in subtype B the prevalence of TDR (160/1,294; 12.4%) was significantly higher than in non-B subtypes (48/591; 8.1%; *p* < 0.01). However, the highest level of TDR (14.9%) was identified in subtype A (Table 3).

### Prevalence of TDR mutations and their impact on drug susceptibility according to the Stanford HIVdb

**Fig 3:**
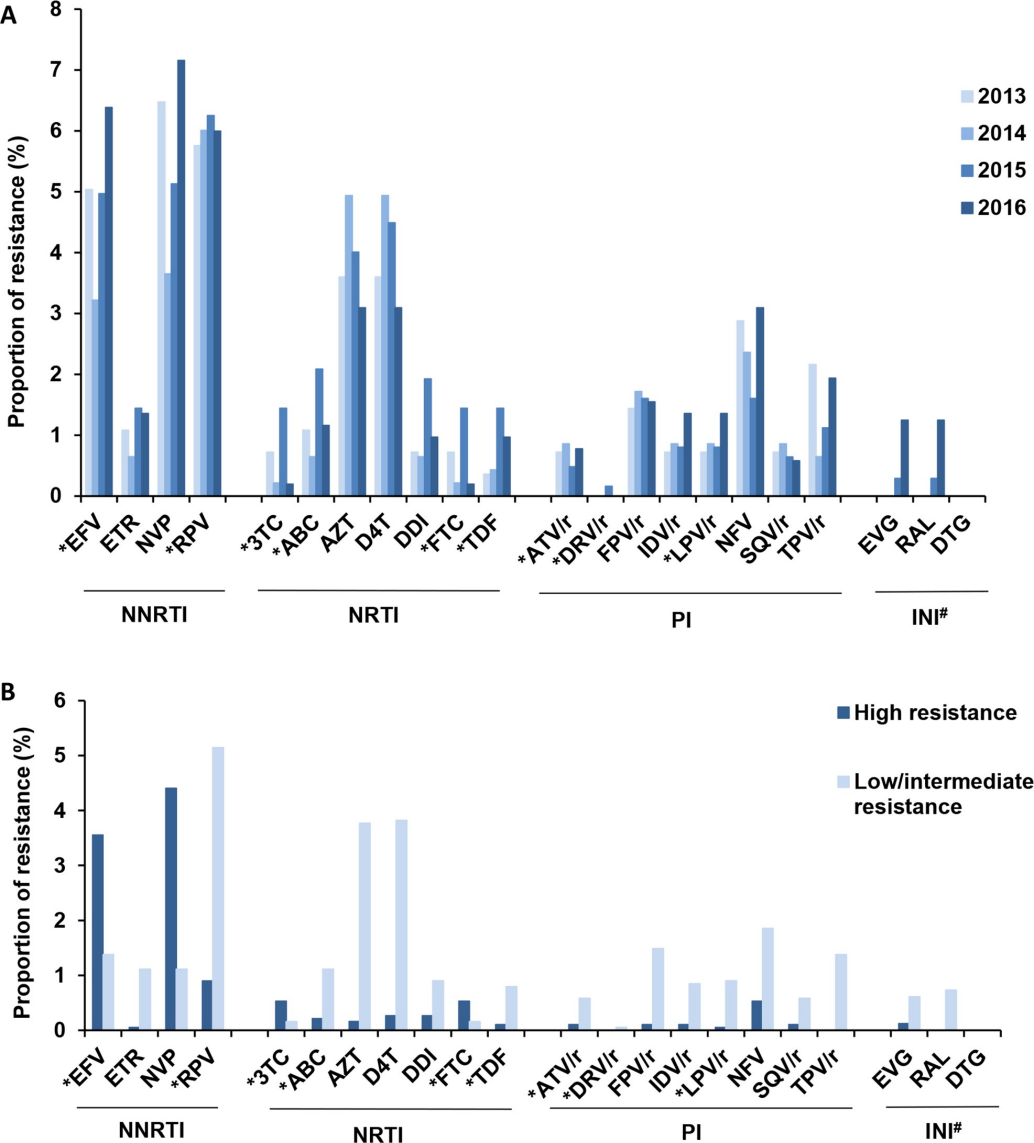
Prevalence of predicted resistance to approved HIV therapeutics according to the Stanford HIVdb. (A) Resistance (low/intermediate/high level) stratified according to the year of diagnosis. (B) Resistance level; # calculated from 820 cases;* drugs included in recommended and alternative first-line treatment option according to EACS 9.0 guidelines; EFV efavirenz, ETR etravirine, NVP nevirapine, RPV rilpivirine, 3TC lamivudine, ABC abacavir, AZT zidovudine, D4T stavudine, DDI didanosine, FTC emtricitabine, TDF tenofovir, ATV atazanavir, DRV darunavir, FPV fosamprenavir, IDV indinavir, LPV lopinavir, NFV nelfinavir, SQV saquinavir, TPV tipranavir, EVG elvitegravir, RAL raltegravir, DTG dolutegravir.

The thymidine analogue mutations (TAMs) M41L, K219Q and T215 revertants as well as the non-TAM M184V were among the most frequently transmitted NRTI-resistance mutations (≥ 0.5%) (Table 4). These TAMs were responsible for the high proportions of resistance to zidovudine and stavudine (both 4%) (Fig 3A) at low/intermediate level (Fig 3B). Less frequently observed were the mutations D67N (n = 8), K65R (n = 2) and K70R (n = 2). The M184V, K70R and K65R mutations induce high level resistance to NRTIs in recommended first-line regimen according to EACS (9.0). However, their abundance was low with 1.3% (abacavir 1.3%, lamivudine 0.7%, tenofovir 0.9% and emtricitabine 0.7%) (Fig 3A).

**Table 4:** Most frequently identified TDR mutations (≥ 0.5%) within the year of diagnosis and indoors HIV-1 subtypes.

	n	2013-2016(n = 1,885) %	2013(n = 278) %	2014(n = 466)%	2015(n = 624)%	2016(n = 517)%	A(n = 154) %	B(n = 1,294)%	C (n = 95)%	CRF02_AG (n = 108) %
**NRTI**										
M41L	21	1.1	1.4	1.5	1.0	0.8	0.6	1.5	-	-
M184V	9	0.5	0.7	0.2	0.8	0.2	0.6	0.3	2.1	0.9
T215rev	49	2.6	1.4	3.0	2.9	2.5	0.6	3.7	-	-
K219Q	12	0.6	0.7	0.6	1.0	0.2	1.3	0.6	1.1	0.9
**NNRTI**										
K103N	57	3.0	3.2	1.7	3.0	4.1	3.2	3.4	3.2	2.8
*V108I	9	0.5	1.4	0.2	0.2	0.6	0.6	0.5	-	-
*E138A	75	4.0	4.7	4.3	3.8	3.5	6.5	3.5	3.2	5.6
**PI**										
M46I/L	28	1.5	2.2	1.3	1.0	1.9	7.8	1.1	1.1	0.9
Q58E	10	0.5	1.4	-	0.3	0.8	-	0.8	-	-
V82L	15	0.8	0.7	0.6	0.8	1.0	-	1.2	-	-
L90M	9	0.5	0.7	0.6	0.5	0.2	-	0.6	-	-

* mutations according to Stanford HIVdb, not listed in the SDRM-list

**Fig 4:**
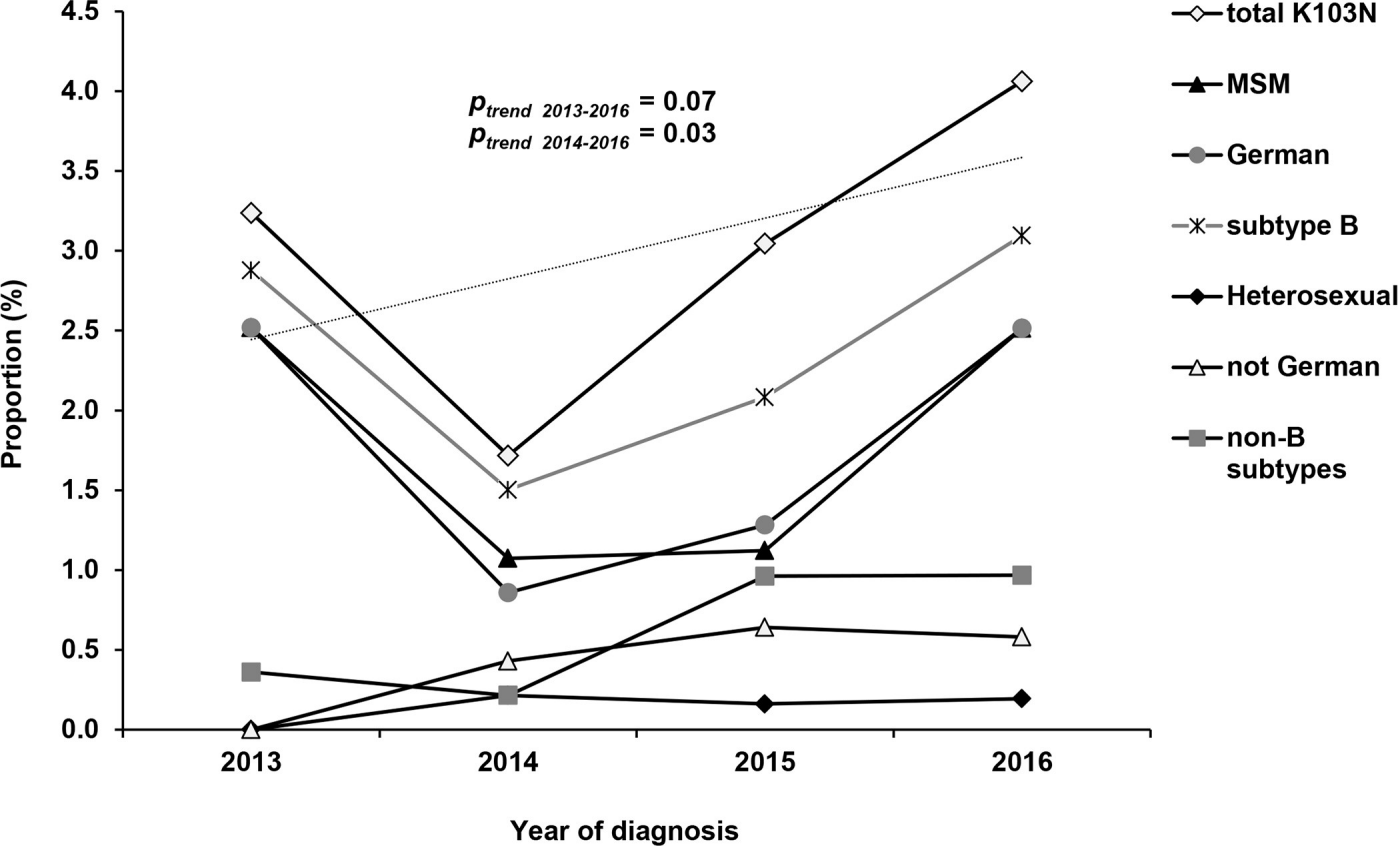
Trends for the K103N mutation between 2013 and 2016 in total and in subgroups. (A) in MSM, persons with German Origin or subtype B infection (B) in persons with heterosexual transmission route, with non-German origin and with subtype non-B infection. Trend line and *p-*value are indicated for proportions of total K103N. MSM: men who have sex with men.

The most frequently detected NNRTI-mutation was K103N. Its proportion was increasing between 2013 and 2016 (*p*_*trend*_ = 0.07) with this increase being significant between 2014 and 2016 (*p*_*trend*_ = 0.03). The steep slope of the `total K103N´ between 2014 and 2016 was quite congruent in the subgroups of MSM, persons of German origin and subtype B infections (Fig 4A). However, slightly increasing tendencies with a peak in 2015 could also be observed for persons with non-German origin and subtype non-B infections (Fig 4B). Consequently, resistance to first generation NNRTIs (efavirenz and nevirapine) doubled between 2014 and 2016 (Fig 3A). The polymorphic NNRTI-mutation E138A not recorded in the SDRM list but associated with low level resistance to the second generation NNRTI rilpivirine according to Stanford HIVdb was found even more frequently during the whole study period (Table 4 and Fig 3B). Therefore, phenotypic resistance to the first-line recommended or alternative NNRTIs (efavirenz 4.9% and rilpivirine 6%) (Fig 3A) was relatively high with 9.1% (172/1,885).

PI-mutations M46I/L and V82L that are responsible for low or intermediate resistance levels to tipranavir, nelfinavir and fosamprenavir (Fig 3B) were also frequently present (Table 4) but without identifiable tendency during the study period (Fig 3A). However, for the first-line recommended or alternative PIs predicted phenotypic resistance was very low with 1% (atazanavir 0.7% and lopinavir 1.0%).

Transmitted INSTI mutations according to Stanford SDRM Worksheet for INSTI were identified in a single sequence among 820 analysed cases (0.12%), namely the major primary mutation T66I resulting in high level resistance to elvitegravir and low level resistance to raltegravir. According to predictions from the Stanford HIVdb, phenotypic INSTI resistance (excluding potential low level) was identified in 0.7% (6/820) of cases (Fig 3B) due to the presence of the T66I (n = 1), the G163R or K (n = 4) or the combination of T97A and E157Q (n = 1) resulting in low level resistance to elvitegravir and raltegravir. These cases were absent in 2014, but exhibited an increase to 0.3% (1/624) in 2015 and to 1.2% (5/517) in 2016 (Fig 3A). None of the sequences showed evidence for resistance to dolutegravir (Fig 3). Taken together, primary resistance to recommended drugs for first-line therapy according to EACS 9.0 was 10.9% (205/1,885).

### Transmission cluster analysis of TDR mutations

**Fig 5:**
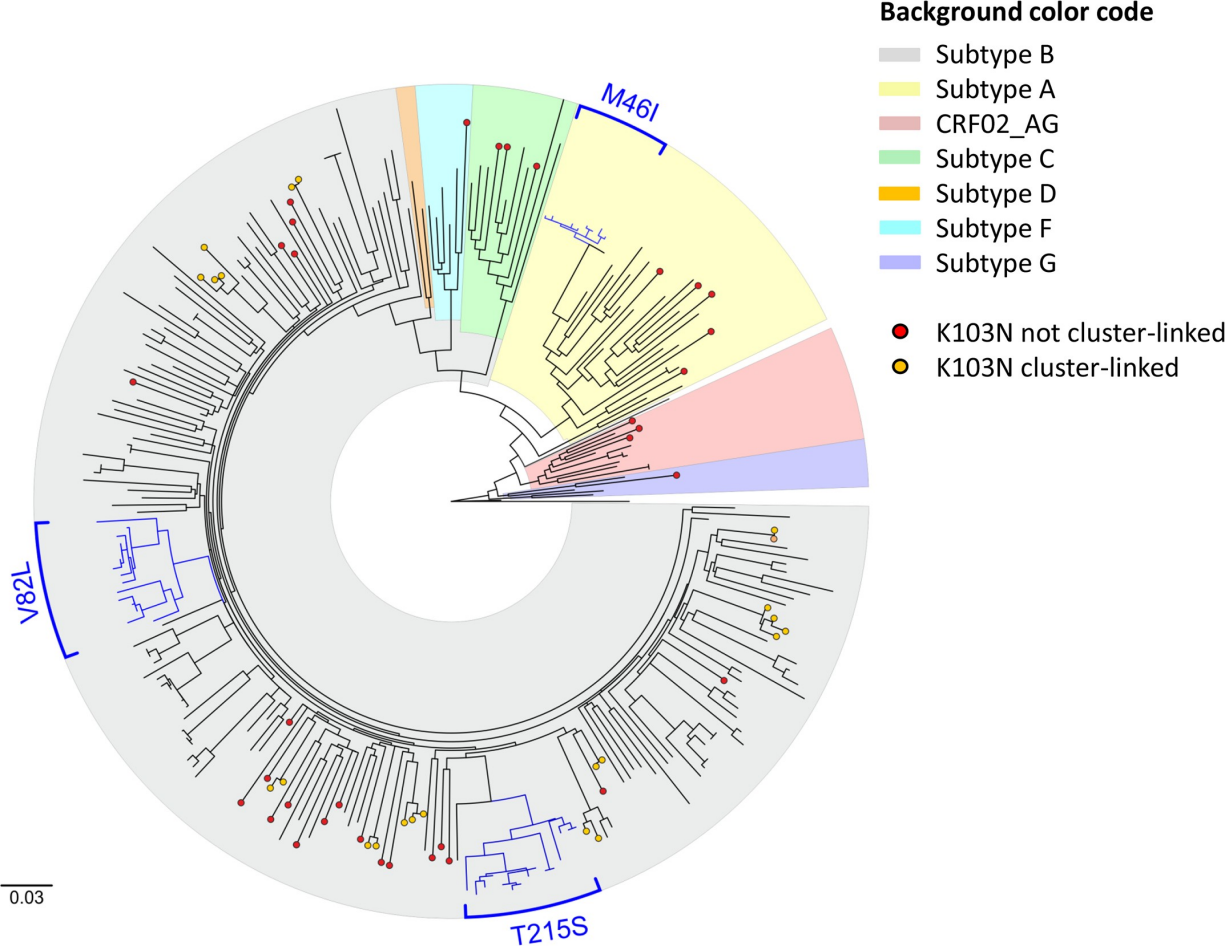
Phylogenetic analysis of 251 HIV-1 sequences carrying drug resistance mutations. Three large clusters with M46I, V82L or T215S mutations are shown with blue branches and all sequences carrying the K103N mutation are depicted with dots at the tips.

Sequences that had one or more of the most frequent NRTI, NNRTI and PI resistance mutations or the polymorphic E138A (listed in Table 4) were selected for phylogenetic analysis (n = 251). All relevant sequence data have been deposited in the NCBI database. All 251 accession numbers are provided within the Supporting Information file, S2 Text. Among them, half were found in one of 32 clusters identified (49.4%, 124/251). The average was 3.9 (min 2 –max 16) sequences per cluster. Three large clusters consisting exclusively of male individuals and mainly MSM and Germans were recognized. Cluster 1 consists of 11 subtype A sequences with the M46I mutation. 15 subtype B sequences with the V82L mutation form cluster 2 and 16 subtype B sequences with the revertant T215S form the third large cluster (Fig 5). The proportion of these major TDR mutations transmitted in clusters and the respective cluster sizes are given in Table 5.

**Table 5:** Prevalence of mutations identified in clusters by phylogenetic analysis of 251 sequences with drug resistance mutations.

Mutation (n)	Proportion in clusters (%)	Cluster with 2 individuals	Cluster with 3–4 individuals	Cluster with 5–7 individuals	Cluster with >7 individuals
M46I (20)	75.0	2	-	-	1 (n = 11)
M46L (8)	37.5	-	1	-	-
V82L (15)	100.0	-	-	-	1 (n = 15)
L90M (9)	33.3	-	1	-	-
Q58E (10)	20.0	1	-	-	-
K103N (57)	40.4	6	3	-	-
V108l (9)	0.0	-	-	-	-
E138A (75)	37.3	2	3	2	-
M41L (21)	42.9	-	1	1	-
M184V (9)	0.0	-	-	-	-
T215E (10)	40.0	2	-	-	-
T215S (31)	80.6	3	1	-	1 (n = 16)
K219Q (12)	58.3	2	1	-	-

**Fig 6:**
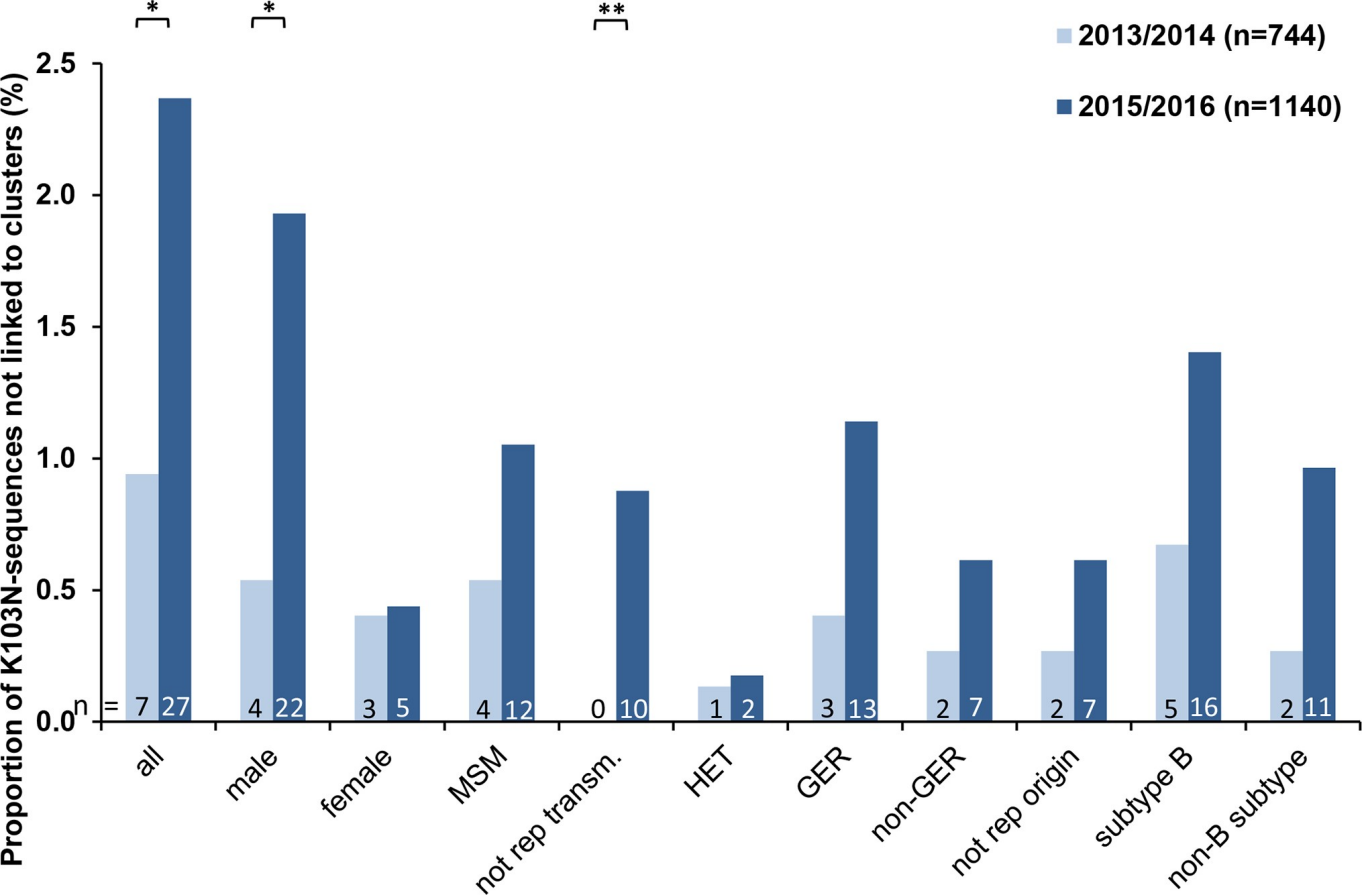
Proportion of not-clusters-linked sequences with K103N mutation according to sub-groups of HIV-infected individuals in 2013/2014 compared to 2015/2016. K103N sequences are grouped according to gender, transmission route, origin and HIV-subtype of the infected person. The proportion is calculated from the total number of sequences of two respective 2-year periods (2013/2014: 744 sequences; 2015/2016: 1140 sequences). Between the two year periods a significant increase of the K103N mutation in males who are not linked to transmission clusters is evident. MSM: men who have sex with men; HET: persons with heterosexual mode of transmission, GER: German, not rep. transm.: not reported transmission, * *p* < 0.05, **p < 0.01.

Special attention was paid to the clustering of the K103N mutation in order to analyse whether the previously described increase in 2015/2016 was the result of a recent spread within one or few active transmission networks and/or within a specific transmission group. Around 40% of the sequences carrying the K103N mutation were found within nine different clusters (all subtype B, cluster size of 2–4 sequences per cluster) (Table 5, Fig 5). Persons in this `cluster-linked´ group were all male, MSM and German or their transmission group and/or origin was not reported. The proportion of individuals within `cluster-linked´ subgroups was quite similar in the years 2013/2014 and 2015/2016 (*p* = 0.67). Among the `not cluster-linked´ sequences carrying the K103N mutation the diversity of HIV-1 subtypes (A, B, C, G, CRF02_AG) as well as the origin of infected persons (Germany, East Europe, West Europe, Latin America, Sub Saharan Africa and not reported) was much higher. The proportion of individuals not linked to K103N-clusters was generally higher in 2015/2016 than in 2013/2014 (*p* = 0.01), although the sampling was more dense in 2015/2016 (n = 1,141 compared to 744 in 2013/2014). Especially the group of `male persons´ and here particularly those for which the transmission route was not reported was significantly higher in 2015/2016 (*p* = 0.008) (Fig 6). However, insignificantly higher proportions were also present in MSM, Germans, non-Germans, persons with unknown origin, infected with subtype B as well as non-B infected but without reaching significance (all *p* > 0.05) (Fig 6).

## Discussion

In this study we analysed 1,885 recent HIV-1 infections newly diagnosed between 2013 and 2016. The proportions of HIV-1 subtypes and primary resistances in the total population and within subgroups (mode of transmission, region of origin) were stratified according to the year of diagnosis to allow analysing changes over time to be.

In Germany, subtype B continues to be the predominant subtype (68.9%), although the proportion of non-B subtypes significantly increased between 2013 and 2016, particularly for subtypes A and CRF02_AG which peaked in 2015 (11.7% and 7.1%) and subtype C which doubled from 3.6% in 2013/2014 [7] to 7.7% in 2016. This increase is partly attributed to persons immigrating from countries where these subtypes are highly prevalent [31] (Sub-Saharan Africa and Central/East Europe) as a result of the flow of refugees into Europe, including Germany with its highest peak in 2015. However, the increase of infections with subtypes A and C was attributed even more to the spread within the German population: high proportions were found in German MSM (subtype A) and German HET and PWID (subtype C). A general increase of non-B subtypes as well as a subtype diversification is a phenomenon that was also observed for many other European countries [32–34].

TDR in Germany analysed by using the WHO surveillance drug resistance list [10] was stable at ~11% between 2013 and 2016, which is in agreement with the data from the German HIV-1 Seroconverter Cohort (11.9% overall TDR in 1996–2010) [35]. TDR at a lower level (8.3%) has also been calculated across 26 European countries for patients diagnosed between 2008 and 2010. TDR in Germany continues to be more frequent among MSM, as reported for other European countries [12, 36] and continues to be significantly more frequent in subtype B than in non-B subtypes. However, the difference appears to be narrowing [37] due to the increase of TDR in non-B subtypes from 6.4% in a previous study [7] to 8.1% in this study. A high proportion of TDR was found in subtype A as a result of one German MSM transmission network spreading the PI resistance mutation M46I. While proportions of NRTI and PI resistance are similar in the present study compared to those reported for Europe [12], the prevalence of NNRTI resistance is about two times higher than previously described for Germany [7] and compared to other countries [12]. This increase was mainly driven by the K103N mutation selected by the first generation NNRTIs nevirapine and efavirenz. While nevirapine was removed from the European treatment guidelines several years ago, efavirenz is still listed as alternative regimen for first-line therapy [5] but is very rarely used in Germany [38]. Therefore, 'de novo' selection of K103N mutations in Germany followed by transmission to newly diagnosed persons seems to be unlikely in times of highly suppressive cART and pre-treatment resistance testing. In contrast, selection of resistance followed by an alarmingly increasing trend of TDR—especially for NNRTI resistances—from regions where first generation NNRTIs are commonly administered due to cost issues (e.g. Sub-Saharan Africa) is reported [39]. Therefore, we assume that the increase of the K103N mutation in our study population is the result of two developments: firstly, the import of the K103N mutation with non-B subtypes from abroad appears to have occurred at quite low levels (Fig 4B). However, the majority of such imported infections with phylogenetically unrelated viruses, even if reported in Germany as a “new diagnosis” in the study period, might not have been analysed here because the vast majority is likely long standing which were excluded from the study.

Secondly, the increasing trend of K103N infections in MSM, individuals of German origin and with subtype B infection (Fig 4A) might be explained by the fact that German MSM are known to have the highest rates of recent infections because they test more frequently than other transmission groups [19]. Although phylogenetic analysis revealed that there was no significant spread of K103N within one or more active transmission networks (as shown for the PI mutations among MSM) we assume that these phylogenetic unrelated K103N carrying strains are a result of continuous onwards transmission of the persisting K103N mutation among long term as well as recent infected individuals and therefore, are not appearing as a coherent transmission network in our phylogenetic analysis. Comprehensive transmission network analysis that includes recent as well as long standing infections might therefore provide some clarification for Germany. Along these lines, increasing proportions of NNRTI resistance and particularly of the K103N mutation were also reported from MSM networks in Greece and in San Diego [40, 41].

Large transmission networks of German MSM were observed for the most frequently transmitted NRTI mutation T215S (revertant of T215Y/F), and the PI resistance mutations M46I and V82L. Nevertheless, the proportions of transmitted NRTI and PI resistance remained stable during the study period. NRTI resistance resulted mainly from the high prevalence of persisting TAMs. Many studies have shown by phylogenetic analysis that onward transmission among drug-naïve patients is the major reason for the maintenance of stable NRTI resistance levels [41–45]. However, as the TAM selecting drugs zidovudine and stavudine have been replaced by tenofovir in current treatment guidelines, TAMs are no longer of direct clinical relevance. The proportion of transmitted PI resistance was higher in Germany (3.2%) than in other European countries (2.0%) [12], presumably due to large clusters of the M46I and V82L mutations. However, their impact on PIs recommended in current first-line therapies remains low.

Therefore, the predicted resistance to the currently recommended first-line regimens [5] consisting of two NRTI plus one PI or one INSTI is very low at the population level (<2.3%) while primary resistance to NNRTI is frequent with 9.1%: 4.9% to efavirence (due to the K103N mutation) and 6.0% to rilpivirine (due to the frequent E138A polymorphism). Similar proportions were reported recently from the UK Drug Resistance Database with 8.2% NNRTI resistance including 6.2% resistance to rilpivirine and 3.4% to efavirenz [36]. Resistance testing is therefore particularly recommended for patients starting with or switching to an NNRTI-containing regimen.

In 2017, tenofovir-containing pre-exposure prophylaxis (PrEP, e.g. tenofovir in combination with emtricitabine) was introduced in Germany. HIV-infection despite PrEP, due to subclinical drug levels or infection with resistant variants, might occur and fuel the emergence of TDR. So far, resistance mutations selected by tenofovir and emtricitabine (K65R, K70R and M184V) have been rare (below 1%). However, as MSM transmission networks have been identified to be a major source for the spread of HIV resistance by others [46] and by us, frequent HIV-screening for PrEP users and the monitoring of PrEP-selected mutations is recommended.

For the first time, we analysed, to which extent INSTI resistance was transmitted following the introduction of the first generation INSTI raltegravir in Germany in 2007. So far, the proportion of transmitted INSTI mutations is low with only one case identified. Phenotypic resistance to raltegravir and elvitegravir according to Stanford HIVdb is predicted to be 0.7% and resistance to the second generation INSTI dolutegravir was not detected at all. Primary drug resistance to INSTIs was also described to be rare in studies of European patients diagnosed in 2013 [36] or 2015 [47]. However, since transmission of INSTI resistance in Germany might occur with a delay the need for follow-up in the future remains.

One limitation of the present study is the relatively short study period. Changes over time should therefore be interpreted carefully. Furthermore, integrase genotyping only started in 2014, resulting in lower PCR success rates due to RNA degradation on DSS. A combined prediction including all drug classes was only possible for 43.0% of cases therefore, resistance to integrase was analysed separately in this dataset. Another limitation is that the SDRM list has not been updated since 2009 and resistance mutations to the newest drugs or drug classes (e.g. integrase inhibitors) had to be analysed with different mutation lists. Some of the SDRMs are only relevant to older drugs rarely used in today’s first-line regimens in EU/EEA countries [4, 5]. If the SDRM list is used, the weighting for TAMs is disproportionately high since they no longer have clinical relevance. On the other hand, NNRTI resistance is underestimated due to the lack of the E138A mutation. The relevance of TDR for the outcome of first-line therapy in EU/EEA countries as calculated from the SDMR list should therefore be interpreted by taking this underestimation into consideration.

## Conclusion

Despite effective cART, TDR is present at an overall stable proportion in Germany (11%). In particular, viruses carrying resistance mutations with low fitness cost are spread continuously by onwards transmission, and German MSM are generally driving the spread in Germany, both within transmission networks and outside of networks as shown for the K103N mutation. Intensified HIV-screening in these groups followed by early treatment with cART including the pre-treatment resistance testing as recommended in the current guidelines should help reduce the spread of resistant viruses in the ART naïve population. Due to the increasing use of INSTIs in first-line regimens and tenofovir/emtricitabin for PrEP, it is important to monitor TDR for public health efforts and in order to maintain the effectiveness of cART.

## Supporting information

S1 Text Validation of the NGS ambiguity threshold. (DOCX)

S2 Text Accession numbers for NCBI database. (DOCX)
