# Supplementary material for: Increasing proportions of HIV-1 non-B subtypes and of NNRTI resistance between 2013 and 2016 in Germany: Results from the national molecular surveillance of new HIV-diagnoses
Source: PLoS One. 2018 Nov 8;13(11):e0206234. doi: 10.1371/journal.pone.0206234 (PMC6224275; doi:10.1371/journal.pone.0206234)
Supplement: S1 Text — (DOCX) [file pone.0206234.s001.docx]

**Supporting information**

**S1 Text. Validation of the NGS ambiguity threshold.**

In total, 399 PR-RT and 177 INT PCR products were sequenced by both Sanger and NGS. Different thresholds for calling ambiguities were then applied to the NGS consensus sequences and compared to the Sanger sequences. The best match between Sanger and NGS sequences was achieved at an ambiguity threshold of 20%. The mean nucleotide sequence identity for the 1026 bp long PR-RT and the 837 bp long INT sequence was 99.8% (range from 95.8% to 100%) and 99.9% (range from 98.9% to 100%), respectively. When counting ambiguous positions including the corresponding nucleotide as match, the mean sequence identity increased to 100% for PR-RT (range from 99.8% to 100%) and 100% for INT (range from 99.9% to 100%). Non-corresponding ambiguities or differing pure nucleotides were detected 17 times in PR-RT and twice in INT sequences. None of these led to a different drug resistance profile according to the Stanford HIV-DB algorithm.
