# Supplementary material for: Increasing proportions of HIV-1 non-B subtypes and of NNRTI resistance between 2013 and 2016 in Germany: Results from the national molecular surveillance of new HIV-diagnoses
Source: PLoS One. 2018 Nov 8;13(11):e0206234. doi: 10.1371/journal.pone.0206234 (PMC6224275; doi:10.1371/journal.pone.0206234)
Supplement: S2 Text — (DOCX) [file pone.0206234.s002.docx]

BankIt2159732 Seq1 MK064253

BankIt2159732 Seq2 MK064254

BankIt2159732 Seq3 MK064255

BankIt2159732 Seq4 MK064256

BankIt2159732 Seq5 MK064257

BankIt2159732 Seq6 MK064258

BankIt2159732 Seq7 MK064259

BankIt2159732 Seq8 MK064260

BankIt2159732 Seq9 MK064261

BankIt2159732 Seq10 MK064262

BankIt2159732 Seq11 MK064263

BankIt2159732 Seq12 MK064264

BankIt2159732 Seq13 MK064265

BankIt2159732 Seq14 MK064266

BankIt2159732 Seq15 MK064267

BankIt2159732 Seq16 MK064268

BankIt2159732 Seq17 MK064269

BankIt2159732 Seq18 MK064270

BankIt2159732 Seq19 MK064271

BankIt2159732 Seq20 MK064272

BankIt2159732 Seq21 MK064273

BankIt2159732 Seq22 MK064274

BankIt2159732 Seq23 MK064275

BankIt2159732 Seq24 MK064276

BankIt2159732 Seq25 MK064277

BankIt2159732 Seq26 MK064278

BankIt2159732 Seq27 MK064279

BankIt2159732 Seq28 MK064280

BankIt2159732 Seq29 MK064281

BankIt2159732 Seq30 MK064282

BankIt2159732 Seq31 MK064283

BankIt2159732 Seq32 MK064284

BankIt2159732 Seq33 MK064285

BankIt2159732 Seq34 MK064286

BankIt2159732 Seq35 MK064287

BankIt2159732 Seq36 MK064288

BankIt2159732 Seq37 MK064289

BankIt2159732 Seq38 MK064290

BankIt2159732 Seq39 MK064291

BankIt2159732 Seq40 MK064292

BankIt2159732 Seq41 MK064293

BankIt2159732 Seq42 MK064294

BankIt2159732 Seq43 MK064295

BankIt2159732 Seq44 MK064296

BankIt2159732 Seq45 MK064297

BankIt2159732 Seq46 MK064298

BankIt2159732 Seq47 MK064299

BankIt2159732 Seq48 MK064300

BankIt2159732 Seq49 MK064301

BankIt2159732 Seq50 MK064302

BankIt2159732 Seq51 MK064303

BankIt2159732 Seq52 MK064304

BankIt2159732 Seq53 MK064305

BankIt2159732 Seq54 MK064306

BankIt2159732 Seq55 MK064307

BankIt2159732 Seq56 MK064308

BankIt2159732 Seq57 MK064309

BankIt2159732 Seq58 MK064310

BankIt2159732 Seq59 MK064311

BankIt2159732 Seq60 MK064312

BankIt2159732 Seq61 MK064313

BankIt2159732 Seq62 MK064314

BankIt2159732 Seq63 MK064315

BankIt2159732 Seq64 MK064316

BankIt2159732 Seq65 MK064317

BankIt2159732 Seq66 MK064318

BankIt2159732 Seq67 MK064319

BankIt2159732 Seq68 MK064320

BankIt2159732 Seq69 MK064321

BankIt2159732 Seq70 MK064322

BankIt2159732 Seq71 MK064323

BankIt2159732 Seq72 MK064324

BankIt2159732 Seq73 MK064325

BankIt2159732 Seq74 MK064326

BankIt2159732 Seq75 MK064327

BankIt2159732 Seq76 MK064328

BankIt2159732 Seq77 MK064329

BankIt2159732 Seq78 MK064330

BankIt2159732 Seq79 MK064331

BankIt2159732 Seq80 MK064332

BankIt2159732 Seq81 MK064333

BankIt2159732 Seq82 MK064334

BankIt2159732 Seq83 MK064335

BankIt2159732 Seq84 MK064336

BankIt2159732 Seq86 MK064337

BankIt2159732 Seq87 MK064338

BankIt2159732 Seq88 MK064339

BankIt2159732 Seq89 MK064340

BankIt2159732 Seq90 MK064341

BankIt2159732 Seq91 MK064342

BankIt2159732 Seq92 MK064343

BankIt2159732 Seq94 MK064344

BankIt2159732 Seq95 MK064345

BankIt2159732 Seq96 MK064346

BankIt2159732 Seq98 MK064347

BankIt2159732 Seq99 MK064348

BankIt2159732 Seq100 MK064349

BankIt2159732 Seq101 MK064350

BankIt2159732 Seq102 MK064351

BankIt2159732 Seq103 MK064352

BankIt2159732 Seq104 MK064353

BankIt2159732 Seq105 MK064354

BankIt2159732 Seq106 MK064355

BankIt2159732 Seq107 MK064356

BankIt2159732 Seq108 MK064357

BankIt2159732 Seq109 MK064358

BankIt2159732 Seq111 MK064359

BankIt2159732 Seq112 MK064360

BankIt2159732 Seq113 MK064361

BankIt2159732 Seq114 MK064362

BankIt2159732 Seq115 MK064363

BankIt2159732 Seq116 MK064364

BankIt2159732 Seq117 MK064365

BankIt2159732 Seq118 MK064366

BankIt2159732 Seq119 MK064367

BankIt2159732 Seq120 MK064368

BankIt2159732 Seq121 MK064369

BankIt2159732 Seq122 MK064370

BankIt2159732 Seq123 MK064371

BankIt2159732 Seq124 MK064372

BankIt2159732 Seq125 MK064373

BankIt2159732 Seq126 MK064374

BankIt2159732 Seq128 MK064375

BankIt2159732 Seq129 MK064376

BankIt2159732 Seq130 MK064377

BankIt2159732 Seq131 MK064378

BankIt2159732 Seq133 MK064379

BankIt2159732 Seq134 MK064380

BankIt2159732 Seq135 MK064381

BankIt2159732 Seq136 MK064382

BankIt2159732 Seq137 MK064383

BankIt2159732 Seq138 MK064384

BankIt2159732 Seq139 MK064385

BankIt2159732 Seq140 MK064386

BankIt2159732 Seq141 MK064387

BankIt2159732 Seq142 MK064388

BankIt2159732 Seq143 MK064389

BankIt2159732 Seq144 MK064390

BankIt2159732 Seq145 MK064391

BankIt2159732 Seq146 MK064392

BankIt2159732 Seq147 MK064393

BankIt2159732 Seq148 MK064394

BankIt2159732 Seq149 MK064395

BankIt2159732 Seq150 MK064396

BankIt2159732 Seq151 MK064397

BankIt2159732 Seq152 MK064398

BankIt2159732 Seq153 MK064399

BankIt2159732 Seq154 MK064400

BankIt2159732 Seq155 MK064401

BankIt2159732 Seq156 MK064402

BankIt2159732 Seq157 MK064403

BankIt2159732 Seq158 MK064404

BankIt2159732 Seq159 MK064405

BankIt2159732 Seq160 MK064406

BankIt2159732 Seq161 MK064407

BankIt2159732 Seq162 MK064408

BankIt2159732 Seq163 MK064409

BankIt2159732 Seq164 MK064410

BankIt2159732 Seq165 MK064411

BankIt2159732 Seq166 MK064412

BankIt2159732 Seq167 MK064413

BankIt2159732 Seq168 MK064414

BankIt2159732 Seq169 MK064415

BankIt2159732 Seq170 MK064416

BankIt2159732 Seq171 MK064417

BankIt2159732 Seq172 MK064418

BankIt2159732 Seq173 MK064419

BankIt2159732 Seq174 MK064420

BankIt2159732 Seq175 MK064421

BankIt2159732 Seq176 MK064422

BankIt2159732 Seq177 MK064423

BankIt2159732 Seq178 MK064424

BankIt2159732 Seq179 MK064425

BankIt2159732 Seq180 MK064426

BankIt2159732 Seq181 MK064427

BankIt2159732 Seq182 MK064428

BankIt2159732 Seq183 MK064429

BankIt2159732 Seq184 MK064430

BankIt2159732 Seq185 MK064431

BankIt2159732 Seq186 MK064432

BankIt2159732 Seq187 MK064433

BankIt2159732 Seq188 MK064434

BankIt2159732 Seq189 MK064435

BankIt2159732 Seq190 MK064436

BankIt2159732 Seq191 MK064437

BankIt2159732 Seq192 MK064438

BankIt2159732 Seq193 MK064439

BankIt2159732 Seq194 MK064440

BankIt2159732 Seq195 MK064441

BankIt2159732 Seq196 MK064442

BankIt2159732 Seq197 MK064443

BankIt2159732 Seq198 MK064444

BankIt2159732 Seq199 MK064445

BankIt2159732 Seq200 MK064446

BankIt2159732 Seq201 MK064447

BankIt2159732 Seq202 MK064448

BankIt2159732 Seq203 MK064449

BankIt2159732 Seq204 MK064450

BankIt2159732 Seq205 MK064451

BankIt2159732 Seq206 MK064452

BankIt2159732 Seq207 MK064453

BankIt2159732 Seq208 MK064454

BankIt2159732 Seq209 MK064455

BankIt2159732 Seq210 MK064456

BankIt2159732 Seq211 MK064457

BankIt2159732 Seq212 MK064458

BankIt2159732 Seq213 MK064459

BankIt2159732 Seq214 MK064460

BankIt2159732 Seq215 MK064461

BankIt2159732 Seq216 MK064462

BankIt2159732 Seq217 MK064463

BankIt2159732 Seq218 MK064464

BankIt2159732 Seq219 MK064465

BankIt2159732 Seq220 MK064466

BankIt2159732 Seq221 MK064467

BankIt2159732 Seq222 MK064468

BankIt2159732 Seq223 MK064469

BankIt2159732 Seq224 MK064470

BankIt2159732 Seq225 MK064471

BankIt2159732 Seq226 MK064472

BankIt2159732 Seq227 MK064473

BankIt2159732 Seq228 MK064474

BankIt2159732 Seq229 MK064475

BankIt2159732 Seq230 MK064476

BankIt2159732 Seq231 MK064477

BankIt2159732 Seq232 MK064478

BankIt2159732 Seq233 MK064479

BankIt2159732 Seq234 MK064480

BankIt2159732 Seq235 MK064481

BankIt2159732 Seq236 MK064482

BankIt2159732 Seq237 MK064483

BankIt2159732 Seq238 MK064484

BankIt2159732 Seq239 MK064485

BankIt2159732 Seq240 MK064486

BankIt2159732 Seq241 MK064487

BankIt2159732 Seq242 MK064488

BankIt2159732 Seq243 MK064489

BankIt2159732 Seq244 MK064490

BankIt2159732 Seq245 MK064491

BankIt2159732 Seq246 MK064492

BankIt2159732 Seq247 MK064493

BankIt2159732 Seq248 MK064494

BankIt2159732 Seq249 MK064495

BankIt2159732 Seq250 MK064496

BankIt2159732 Seq251 MK064497

Sequit 15-Mar-2018_Seq85 MF124727

Sequit 15-Mar-2019_Seq93 MF124734

Sequit 15-Mar-2020_Seq97 MF124741

Sequit 15-Mar-2021_Seq110 MF124753

Sequit 15-Mar-2022_Seq127 MF124780

Sequit 15-Mar-2023_Seq132 MF124787
